# Comparison of Albumin and Ischemia-Modified Albumin Levels in Concurrent Blood and Cerebrospinal Fluid in Patients with Spontaneous Subarachnoid Hemorrhage and Normal Pressure Hydrocephalus

**DOI:** 10.3390/medicina62050954

**Published:** 2026-05-13

**Authors:** Onur Bologur, Huseyin Berk Benek, Hakan Yilmaz, Cafer Ak, Alper Tabanli, Engin Kayikci, Alaettin Yurt

**Affiliations:** Department of Neurosurgery, Izmir Faculty of Medicine, University of Health Sciences, 35170 Izmir, Turkey; bologur@hotmail.com (O.B.); benekberk@gmail.com (H.B.B.); slawerkarwyn@hotmail.com (C.A.); alper_tabanli@hotmail.com (A.T.); enginkayikci@hotmail.com (E.K.);

**Keywords:** biomarker, ischemia-modified albumin, cerebral aneurysm, spontaneous subarachnoid hemorrhage, vasospazm

## Abstract

*Background and Objectives*: Ischemia-modified albumin (IMA) has previously been identified as a biomarker for early ischemia, rapidly formed by acidosis and free radical modification of the N-terminus of human serum albumin. This study aimed to compare albumin and IMA levels in blood and cerebrospinal fluid (CSF) from 30 patients with spontaneous subarachnoid hemorrhage (SAH) and 15 patients with normal pressure hydrocephalus (NPH) at a single center between 2021 and 2022. *Materials and methods*: This prospective study included 30 patients diagnosed with subarachnoid hemorrhage (SAH), confirmed radiologically, who were admitted to the Health Sciences University İzmir Bozyaka Training and Research Hospital and constituted the study group. The control group consisted of 15 patients diagnosed with normal pressure hydrocephalus (NPH) without a history or radiological evidence of subarachnoid hemorrhage or any other intracranial hemorrhagic pathology. In the control group, no pathological findings suggestive of hemorrhage or inflammation were detected in serum or cerebrospinal fluid (CSF) analyses. Blood and CSF samples were collected simultaneously from all participants, and albumin and ischemia-modified albumin (IMA) levels were measured. Serum and CSF albumin and IMA levels were compared between the study and control groups. *Results*: Of the 30 patients included in the study, 19 (63.3%) were male and 11 (36.7%) were female. The albumin level was lower in the patient group compared to the NPH group (3.8 g/dL [1.8–4.7] vs. 4.3 g/dL [3.2–5.0], respectively, *p* = 0.008). The serum IMA level was higher in the patient group compared to the NPH group (0.36 ABSU [0.30–0.65] vs. 0.25 ABSU [0.05–0.32], respectively, *p* = 0.010). The serum IMA level was higher in the vasospasm group compared to the group without vasospasm. *Conclusions*: In patients with SAH, a condition associated with high morbidity and mortality, modified albumin levels were found to be significantly higher in both CSF and blood compared to the NPH group. IMA may be a potential biomarker associated with SAH and vasospasm; however, further large-scale studies with multivariable analysis and external validation are required to confirm its diagnostic and prognostic utility.

## 1. Introduction

SAH can occur after trauma, as a result of rupture of an intracranial aneurysm, due to other pathologies of the cerebral vascular vessels or due to blood leaking from venous structures without any vascular pathology. The present cohort focuses on spontaneous/non-traumatic SAH. The most common cause of spontaneous non-traumatic SAH is aneurysm rupture [1]. It represents roughly 5% of all strokes, with an incidence of about 10.3 per 100,000 person-years, often peaking between ages 50 and 60 [2]. It is characterized by the sudden onset of a severe, maximum-intensity headache (“thunderclap headache”). SAH is diagnosed within the first 6–12 h with a highly sensitive, non-contrast brain CT scan. If the CT scan is negative for SAH but clinical suspicion is high, a lumbar puncture is performed to detect xanthochromia or a high red blood cell count in the CSF. Angiography (CT angiography or DSA) is used to identify a ruptured aneurysm. The main cause of death and disability among patients who survive an aneurysm rupture is the narrowing of the great cerebral veins, leading to delayed cerebral ischemia. Patients who experience spontaneous SAH and survive may develop delayed brain injury through different mechanisms, and patients who survive the acute phase remain at risk of mortality and morbidity due to delayed brain damage. SAH is an important health problem due to the decreased quality of life of patients. It also causes loss of labor due to the peak age of onset in the fifth decade. As disease severity increases, the costs incurred increase. Complications that develop after SAH also increase the economic burden [3,4,5]. Normal pressure hydrocephaly is characterized by the triad of gait instability, urinary incontinence, and dementia. Incidence increases with age. Diagnosis involves MRI/CT showing ventriculomegaly (Evans index > 0.3), confirmed by high-volume lumbar puncture. Treatment is generally surgical CSF shunting.

IMA is a type of altered albumin resulting from exposure to hypoxia, acidosis, inflammation, or oxidative stress. Albumin is one of the most highly concentrated proteins in the bloodstream. In ischemic conditions, it undergoes a series of modifications, resulting in the formation of IMA. Oxygen radicals created by oxidative stress are thought to play a part in the formation of IMA. It has a lower binding capacity for nickel and cobalt than its parent molecule albumin [6]. SAH is an important health problem due to high mortality rates in the acute phase and neurological deterioration that occurs as a result of complications. However, there is still no biomarker routinely used in the treatment and prognosis of patients with SAH. Studies have shown that serum IMA levels may increase in acute ischemic stroke, intracerebral hemorrhage, and SAH [7,8,9,10]. In the present study, IMA levels in serum and CSF were studied in patients with spontaneous SAH and in the NPH group. The aim of the study was to evaluate concurrent IMA levels in serum and CSF in patients with spontaneous SAH and to assess the usefulness of IMA as a biomarker for severity of vasospasm signals.

## 2. Materıals and Methods

### 2.1. Ethics Approval and Consent to Participate

Approval for this study was obtained from the Clinical Research Ethics Committee of Health Sciences University İzmir Bozyaka Training and Research Hospital on 9 June 2021 (Decision No: 2021/99). Written informed consent was obtained from all participants or their legal representatives before enrollment. All procedures were conducted in accordance with the ethical standards of the World Medical Association and the principles of the Declaration of Helsinki.

### 2.2. Study Population

This prospective single-center study was conducted at İzmir Bozyaka Training and Research Hospital between 2021 and 2022. A total of 45 participants were included in the study. The study group consisted of 30 consecutive adult patients diagnosed with spontaneous aneurysmal subarachnoid hemorrhage (aSAH), confirmed by computed tomography (CT) angiography, who presented within 48 h after ictus.

The control group consisted of 15 patients diagnosed with normal pressure hydrocephalus (NPH) who underwent lumbar puncture for diagnostic purposes and had no history or radiological evidence of subarachnoid hemorrhage or any other intracranial hemorrhagic pathology. In addition, no pathological findings suggestive of hemorrhage or inflammation were detected in their serum or cerebrospinal fluid (CSF) analyses.

The inclusion criteria were age ≥18 years and radiologically confirmed aSAH. Exclusion criteria included traumatic subarachnoid hemorrhage, chronic inflammatory diseases, autoimmune diseases, previous ischemic heart disease, previous cerebrovascular ischemic disease, and refusal to participate.

Demographic and clinical data, including age, sex, comorbidities, presenting symptoms, CSF appearance, serum albumin levels, CSF albumin levels, serum ischemia-modified albumin (IMA) levels, and CSF IMA levels, were recorded.

### 2.3. Radiological Evaluation

Admission CT scans were analyzed in detail. Clinical and radiological severity of SAH was classified according to the Hunt–Hess, Fisher, Yaşargil, Botterell, and World Federation of Neurosurgical Societies grading systems. In addition, Glasgow Coma Scale scores were recorded.

Diagnostic CT angiography and magnetic resonance angiography (MRA) findings were reviewed to determine cerebrovascular pathologies. Vasospasm was diagnosed based on transcranial Doppler (TCD) ultrasonography and CT angiography findings. In the TCD evaluation, a ratio of middle cerebral artery flow velocity to extracranial internal carotid artery flow velocity greater than 3 was accepted as diagnostic for vasospasm. Arterial narrowing on cerebral angiography accompanied by delayed contrast filling was also considered indicative of vasospasm.

### 2.4. Laboratory Analysis

Blood and CSF samples were collected simultaneously within 6 h of admission. Blood samples were obtained during routine venous blood sampling procedures performed during hospitalization. CSF samples were collected simultaneously with those obtained for diagnostic purposes during routine clinical follow-up, and no additional invasive procedure was performed for the study.

Venous blood samples were collected in 5 mL vacuum serum separator tubes (Vacusera Serum Clot Activator Tubes, DISERA A.Ş., Istanbul, Turkey). Blood samples were allowed to clot for 30 min and centrifuged at 3000 rpm for 10 min. CSF samples were centrifuged in 5 mL glass tubes at 2000 rpm for 15 min. The obtained serum and CSF supernatants were transferred into 1.5 mL Eppendorf tubes using an automatic pipette and stored at −80 °C until analysis.

Serum albumin levels were measured using an automated colorimetric assay on the Cobas 8000 modular analyzer series (c502 module; Roche Diagnostics, Mannheim, Germany). CSF albumin levels were measured using the Roche immunoturbidimetric albumin assay on the same analyzer.

Serum and CSF IMA levels were measured using the colorimetric cobalt-binding assay described by Bar-Or, with absorbance measured at 470 nm using the Multiskan GO ELISA reader (Thermo Fisher Scientific, Waltham, MA, USA). This method quantifies free cobalt remaining unbound after albumin–cobalt binding.

To eliminate the effect of interindividual differences in albumin concentration on IMA measurements, IMA (IMA) values were calculated using the following formula:

IMA = (individual albumin concentration/median albumin concentration of the study population) × IMA

Serum and CSF IMA levels were expressed as absorbance units (ABSU).

### 2.5. Statistical Analysis

Analyses were performed using SPSS software version 22.0 (IBM, New York, NY, USA). Continuous variables were first evaluated using descriptive statistics. The normality of data distribution was assessed using the Shapiro–Wilk and Kolmogorov–Smirnov tests (Table 1 and Table 2). For univariate comparisons between the SAH and NPH groups, the Student’s *t*-test was used for normally distributed variables, and the Mann–Whitney U test was applied for non-normally distributed variables (Table 3). Categorical variables were compared using the Chi-square test or Fisher’s exact test, as appropriate (Table 4).

To evaluate whether the association between biomarker levels (IMA) and SAH or vasospasm was independent of potential confounding factors, multivariable logistic regression analyses were additionally performed. The models were adjusted for age, sex, comorbidities, and serum albumin levels, as well as SAH severity indicators (when applicable). Results were reported as odds ratios (OR) with 95% confidence intervals (CI). A *p*-value of <0.05 was considered statistically significant.

## 3. Results

### Baseline Characteristics and Clinical Data

The study included 30 patients with SAH (66.7%) and 15 NPH patients (33.3%). The participants’ mean age was 53.8 ± 13.4 years (range 17–86). Of the study participants, 23 (51.1%) were male, and 22 (48.9%) were female. A total of 16 patients (35.6%) had comorbidities (Table 1).

When the patient group and the NPH group were analyzed, the complaints at presentation were headache in 14 (31.1%) patients, altered consciousness in 14 (31.1%) patients, low back pain in nine (20%) patients, trauma in three (6.7%) patients (concomitant injury after SAH-related collapse/fall; traumatic SAH excluded), neck pain in two (4.4%) patients, loss of balance in one (2.2%) patient, loss of strength in one (2.2%) patient, and speech disorder in one (2.2%) patient.

**Figure 1:**
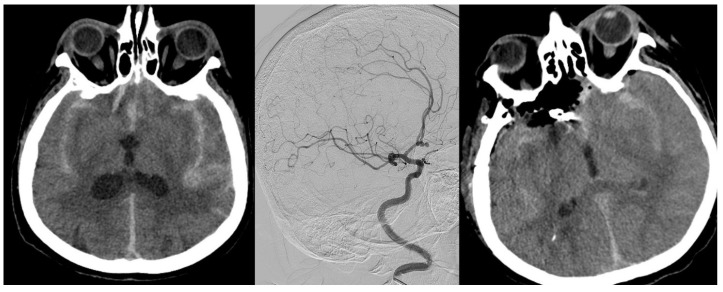
Serum and CSF IMA values were measured in a sample patient with subarachnoid hemorrhage (surgical treatment was performed due to anterior communicating artery aneurysm detected on digital subtraction angiography following subarachnoid hemorrhage).

The mean GCS at admission was 13.6 ± 2.7 (range 4–15). Clinical and radiological characteristics of the patient and control groups were recorded, and WFNS, Hunt–Hess, Yaşargil, and Fisher SAH classification scores were calculated. Since the control group did not have SAH, their scores could not be calculated but were included in the total number. According to the WFNS classification, 10 (22.2%) patients were Stage 1, 13 (28.9%) were Stage 2, one (2.2%) was Stage 3, five (11.1%) were Stage 4, and one (2.2%) was Stage 5. According to the Hunt–Hess classification, six (13.3%) patients were Stage 1, 15 (33.3%) were Stage 2, six (13.3%) were Stage 3, and three (6.7%) were Stage 4. According to the Yaşargil classification, five (11.1%) patients were Stage 0A, four (8.9%) were Stage 1A, 10 (22.2%) were Stage 2A, one (2.2%) was Stage 2B, four (8.9%) were Stage 3A, four (8.9%) were Stage 3B, and two (2.2%) were Stage 4. According to the Fisher classification, four (8.9%) patients were Stage 1, five (11.1%) were Stage 2, six (13.3%) were Stage 3, and 15 (33.3%) patients were Stage 4 (Figure 1).

Hemorrhage was detected on CT in 24 (53.3%) patients, no hemorrhage was detected in 18 (40%) patients, and three (6.7%) patients were suspected of having SAH.

Serum and CSF IMA levels: Comparison between the subarachnoid group and the NPH group. The mean serum albumin level among all 45 participants was 4 ± 0.7 (min: 1.83, max: 4.98). The mean CSF IMA level was 3 ± 4.9 (min: 0.04, max: 26.83), and the mean serum IMA level was 0.3 ± 0.1 (min: 0.05, max: 0.65) (Table 2).

Nineteen (63.3%) of the patients were male, and 11 (36.7%) were female. In the NPH group, four (26.7%) participants were male, and 11 (73.3%) were female. A significant difference was found between the groups in terms of gender (*p* = 0.020); 12 (40%) patients and four (26.7%) in the NPH group had comorbidities, and no significant difference was found between the groups in terms of comorbidities (*p* = 0.378).

The mean GCS score of the patient group was 14 (min: 4, max: 15), while that of the NPH group was 15 (min: 13, max: 15). When compared statistically, GCS scores were significantly lower in the patient group (*p* = 001).

The albumin level was lower in the patient group compared to the NPH group (3.8 [1.8–4.7] vs. 4.3 [3.2–5.0], respectively, *p* = 0.008). This difference was statistically significant.

The serum IMA level was higher in the patient group compared to the NPH group (0.36 [0.30–0.65] vs. 0.25 [0.05–0.32], respectively, *p* = 0.010). This difference was also statistically significant. The CSF IMA level was significantly higher in the patient group compared to the NPH group (1.60 [0.04–26.83] vs. 0.44 [0.18–2.21], respectively, *p* = 0.010) (Table 3). When serum IMA levels were evaluated according to gender in the subarachnoid hemorrhage and normal pressure hydrocephalus groups, no statistically significant difference was found.

In the subarachnoid hemorrhage group, increasing Hunt–Hess scores were associated with a significant decrease in albumin levels and a significant increase in ischemia-modified albumin (IMA) levels. Among patients with subarachnoid hemorrhage, angiographically confirmed vasospasm following clinical deterioration was detected in 8 (26.6%) of 30 patients. In patients who developed vasospasm, albumin levels were significantly decreased, whereas IMA levels were significantly increased in both cerebrospinal fluid and blood samples. The albumin level was lower in the vasospasm group compared to the group without vasospasm (2.4 vs. 3.9, respectively, *p* = 0.004). This difference was statistically significant. The serum IMA level was higher in the vasospasm group compared to the group without vasospasm (0.5 vs. 0.3, respectively, *p* = 0.001). This difference was also statistically significant. The CSF IMA level was significantly higher in the vasospasm group compared to the group without vasospasm (7.5 vs. 2.1, respectively, *p* = 0.04). These findings suggest that albumin and IMA levels may serve as potential biomarkers for predicting the development of vasospasm (Table 4). Considering the small vasospasm subgroup (n = 8), this can be considered a limitation of our study for statistical results.

**Figure 2:**
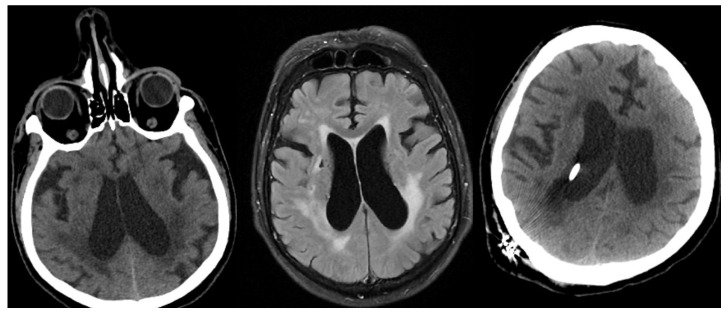
Serum and CSF IMA values were measured in a patient who underwent surgical treatment for normal pressure hydrocephalus.

Aneurysm location was as follows: 12 patients had anterior communicating artery (ACom) aneurysms, eight had middle cerebral artery (MCA) aneurysms, six had internal carotid artery (ICA) aneurysms, and two had posterior circulation aneurysms. In addition, two patients were diagnosed with non-aneurysmal subarachnoid hemorrhage. Regarding treatment modality, 18 patients underwent surgical clipping, while 10 patients were treated with endovascular coiling. During clinical follow-up, external ventricular drainage (EVD) was required in 18 patients (Table 5 and Figure 2).

In the subarachnoid hemorrhage group, increasing Hunt–Hess scores were associated with a significant decrease in albumin levels and a significant increase in ischemia-modified albumin (IMA) levels. Among patients with subarachnoid hemorrhage, angiographically confirmed vasospasm following clinical deterioration was detected in 8 (26.6%) of 30 patients.

In patients who developed vasospasm, albumin levels were significantly lower, whereas IMA levels were significantly higher in both cerebrospinal fluid and serum samples. The albumin level was lower in the vasospasm group compared to the non-vasospasm group (2.4 vs. 3.9, respectively; *p* = 0.004). This difference was statistically significant.

The serum IMA level was higher in the vasospasm group compared to the non-vasospasm group (0.5 vs. 0.3, respectively; *p* = 0.001). This difference was also statistically significant. The CSF IMA level was significantly higher in the vasospasm group compared to the non-vasospasm group (7.5 vs. 2.1, respectively; *p* = 0.04).

These findings indicate a significant association between albumin and IMA levels and the presence of vasospasm. However, due to the small sample size of the vasospasm subgroup (n = 8) and the timing of sample collection within 6 h of admission—prior to the typical development window of vasospasm—these results should be interpreted as exploratory and non-predictive.

## 4. Discussion

In this study, we observed that the IMA value was significantly elevated in patients with subarachnoid hemorrhage, and its elevation correlated with the severity of vasospasm. Significantly higher IMA values were detected in serum and CSF samples compared to the NPH group.

Spontaneous SAH has a high morbidity and mortality rate, and it is most commonly caused by cerebral aneurysms [1,11]. The formation and developmental stages of cerebral aneurysms have not been clearly defined. It is an important public health problem due to the high mortality rates at the time of bleeding and mortality due to the complications and neurological sequelae that occur. Currently, no biomarker is routinely used for the follow-up of patients after SAH. Ischemia plays an important role in the pathophysiological processes of bleeding and the subsequent complications. In the present study, we examined the usefulness of IMA as a biomarker in patients with SAH. We found significantly higher levels of IMA in serum and CSF in patients with SAH.

Various biomarkers have been examined in serum for disease diagnosis, diagnosis of complications, and prognostic evaluation in patients with SAH [12,13]. None of these studies have revealed a biomarker approved by the medical community. These studies need to be validated by further, more comprehensive studies. Studies on CSF are very limited, but there are studies comparing inflammatory and infective outcomes after aneurysmal subarachnoid hemorrhage at the molecular level in CSF, as well as a small number of studies measuring the levels of certain molecules in CSF in patients with SAH [14,15].

IMA has been investigated for its usability as a biomarker in various diseases that involve ischemic processes in their pathophysiology. In 2000, IMA was recommended to be used in the early diagnosis of acute coronary syndromes [16]. In addition, increased IMA levels have been reported in patients after acute aortic dissection compared to healthy individuals [17]. Gündüz et al. showed that IMA levels increased 1.6-fold in ischemic stroke compared to healthy individuals [7]. It is also shown that IMA levels were significantly increased in patients with type 1 and type 2 diabetes mellitus, concluding that IMA can be used as a biomarker [18,19,20].

Another study showed that IMA levels increase in spontaneous abortions in the early stages of pregnancy [21]. In addition, various studies have found elevated IMA levels in patients with various cancers [22,23].

In 2022, a meta-analysis reviewed 17 studies evaluating serum IMA levels in ischemic stroke, intracerebral hemorrhage, and SAH. IMA levels were significantly higher in acute ischemic stroke and intracerebral hemorrhage (*p* < 0.001 and *p* = 0.004, respectively). In patients with SAH, high IMA levels were evaluated to have very low certainty of evidence (*p* = 0.014) [24].

A study examining 50 patients with ischemic cerebrovascular disease showed that their IMA levels were statistically significantly elevated and correlated with increased vasospasm [24,25]. Similarly, in our study, IMA values increased in correlation with the severity of vasospasm in patients with subarachnoid hemorrhage.

Among the 17 studies included in this meta-analysis, IMA levels were measured in only three studies in 90 patients with SAH and 218 controls. IMA levels were significantly higher in patients with SAH compared to the control group (*p* = 0.014). IMA levels were significantly higher in patients with acute ischemic stroke compared to patients with SAH (*p* < 0.001), but not significantly lower compared to patients with intracerebral hemorrhage (*p* = 0.086). According to the results of the analysis, high IMA levels in patients with SAH were expressed as a very low certainty of evidence.

In their study published in 2008, Gündüz et al. compared serum IMA levels in 43 patients with cerebral infarction, 52 patients with SAH, 11 patients with intracerebral hemorrhage, and 43 controls. Accordingly, the serum IMA level was 0.280 ± 0.045 ABSU in patients with cerebral infarction, 0.259 ± 0.053 ABSU in patients with intracerebral hemorrhage, and 0.243 ± 0.061 ABSU in patients with SAH [7]. Consistent with our study, these findings showed high IMA levels in patients with SAH.

In another study, Han et al. (2012) examined blood lipids and serum IMA levels in 62 patients with cerebral infarction, 40 patients with intracerebral hemorrhage, and 18 patients with SAH [8]. Serum IMA levels were 80.81 ± 11.97 U/mL in patients with cerebral infarction, 80.25 ± 10.91 U/mL in patients with intracerebral hemorrhage, 74.43 ± 11.39 U/mL in patients with SAH, and 41.08 ± 5.10 U/mL in the control group. Serum IMA levels were found to be significantly higher in all groups compared to the NPH group (*p* < 0.05) [8].

Elshony et al. evaluated serum fibulin-5 and IMA levels in 50 patients with cerebral ischemia, 30 patients with intracerebral hemorrhage, 20 patients with SAH, and 75 controls. Serum fibulin-5 and IMA levels were found to be significantly higher in the stroke group of 100 patients compared to the control group (*p* < 0.001) [10].

SAH is a condition that occurs in the productive periods of life and has serious consequences. The management of neurological conditions caused by the disease and its complications places a significant burden on the healthcare system. Managing this condition is challenging because of the complications that can occur after hemorrhage. Since the diagnosis is made with imaging methods in practice, it precipitates an increased need for imaging tools. In centers where imaging tools are not available, there may be delays in diagnosis and treatment. There is no biomarker used in routine practice for the diagnosis of SAH. NPH involves specific CSF molecular changes, characterized by reduced levels of Alzheimer’s-related markers and increased markers of neuronal/ependymal damage, such as neurofilament light, vimentin, and inflammatory cytokines like MCP-1 and TNF-α [8]. Since significant IMA changes and vasospasm in NPH are not reported in the literature, they were selected as the comparison group in our study.

Furthermore, the number of biomarker studies in serum and CSF for diagnosis and treatment is limited, and the results are insufficient. In the present study, we found that IMA levels in serum and CSF were significantly higher in patients with spontaneous SAH compared to the NPH group. Therefore, we posit that IMA can be a candidate biomarker in patients with spontaneous SAH. However, it has been shown that IMA levels are elevated in many diseases. The patients in the present study were not examined for comorbidities that could increase IMA levels. This may have affected our results.

In patients with spontaneous SAH, the treatment and management of complications, in addition to the treatment of the primary pathology, are also important.

It is still unclear which patients develop complications and which do not, and the factors affecting patients’ responses to the treatment of complications have not been elucidated. There is no known biomarker that can give information about the prognosis of these patients. Further, more comprehensive studies on spontaneous SAH are needed, and clinicians need extensive information on the management of this disease in clinical practice.

The limitation of the study is that we used patients with normal pressure hydrocephalus as the control group. The control group was randomly selected from among patients with normal pressure hydrocephalus who underwent lumbar puncture due to their symptoms, but in whom no pathology was detected in the serum or cerebrospinal fluid at our clinic. IMA may be a severity marker and not vasospasm because samples were collected within 6 h, and vasospasm is known to occur days later, or even weeks later. So, temporal causality is unclear.

## 5. Conclusions

The present cohort represents spontaneous/non-traumatic SAH. In patients with SAH, a condition with high morbidity and mortality, modified albumin levels were significantly higher in both CSF and blood compared to the NPH group. IMA is a potential candidate biomarker warranting further study.

## Figures and Tables

**Table 1:** Demographic characteristics and comorbidities of the participants.

Age (years) Mean ± SD (Min–Max)	53.8 ± 13.4 (17.0–86.0)
Group n (%)	
Patient	30 (66.7)
Control	15 (33.3)
Gender n (%)	
Male	23 (51.1)
Female	22 (48.9)
Comorbidity n (%)	16 (35.6)

**Table 2:** Serum albumin, serum IMA, and CSF IMA levels of all study participants (descriptive statistics tests, Shapiro–Wilk and Kolmogorov–Smirnov normality tests).

Albumin mean ± SD (min–max)	4.0 ± 0.7 (1.83–4.98)
CSF IMA mean ± SD (min–max)	3.0 ± 4.9 (0.04–26.83)
Serum IMA mean ± SD (min–max)	0.3 ± 0.1 (0.05–0.65)

**Table 3:** Statistical analysis of serum albumin, serum IMA, and CSF IMA levels of the study participants (Student’s *t*-test, Mann–Whitney U test).

	Patients, n = 30	Controls, n = 15	*p* Value
Albumin median (min–max)	3.8 (1.8–4.7)	4.3 (3.2–5.0)	0.008
CSF IMA median (min–max)	1.60 (0.04–26.83)	0.44 (0.18–2.21)	0.010
Serum IMA median (min–max)	0.36 (0.30–0.65)	0.25 (0.05–0.32)	0.001

**Table 4:** Comparison of albumin and IMA levels between vasospasm and non-vasospasm groups (Chi-square and Fisher’s exact test). (Albumin: g/dL; IMA: ABSU).

Parameter	Vasospasm (n = 8)	No Vasospasm (n = 22)	*p*-Value
Albumin level	2.4	3.9	0.004
Serum IMA level	0.5	0.3	0.001
CSF IMA level	7.5	2.1	0.04

**Table 5:** Treatment modality percentages are calculated among aneurysmal SAH patients (n = 28), excluding non-aneurysmal cases.

Variable	n	%
Aneurysm location		
Anterior communicating artery (ACom)	12	40.0
Middle cerebral artery (MCA)	8	26.7
Internal carotid artery (ICA)	6	20.0
Posterior circulation	2	6.7
Non-aneurysmal SAH	2	6.7
Treatment modality		
Surgical clipping	18	62.1
Endovascular coiling	10	34.5
External ventricular drainage (EVD)	18	60.0

## Data Availability

The data that support the findings of this study are available from the corresponding author, upon reasonable request.
